# Cell Wall Properties Determine Genotype-Specific Response to Cold in *Miscanthus* × *giganteus* Plants

**DOI:** 10.3390/cells11030547

**Published:** 2022-02-04

**Authors:** Anna Bilska-Kos, Aleksandra Pietrusińska, Szymon Suski, Agnieszka Niedziela, Anna M. Linkiewicz, Włodzimierz Majtkowski, Grzegorz Żurek, Jacek Zebrowski

**Affiliations:** 1 Department of Biochemistry and Biotechnology, Plant Breeding and Acclimatization Institute—National Research Institute, Radzików, 05-870 Błonie, Poland; a.niedziela@ihar.edu.pl; 2 National Centre for Plant Genetic Resources, Plant Breeding and Acclimatization Institute—National Research Institute, Radzików, 05-870 Błonie, Poland; a.pietrusinska@ihar.edu.pl; 3 Laboratory of Electron Microscopy, Nencki Institute of Experimental Biology of Polish Academy of Sciences, 3 Pasteur, 02-093 Warsaw, Poland; soosky@o2.pl; 4 Molecular Biology and Genetics Department, Institute of Biological Sciences, Faculty of Biology and Environmental Sciences, Cardinal Stefan Wyszyński University, Wóycickiego 1/3, 01-938 Warsaw, Poland; a.linkiewicz@uksw.edu.pl; 5 Genetically Modified Organisms Controlling Laboratory, Plant Breeding and Acclimatization Institute—National Research Institute, Radzików, 05-870 Błonie, Poland; 6 Botanical Garden, National Centre for Plant Genetic Resources, Plant Breeding and Acclimatization Institute—National Research Institute, Jeździecka 5, 85-867 Bydgoszcz, Poland; w.majtkowski@ihar.edu.pl; 7 Department of Bioenergetics, Quality Analysis and Seed Science, Plant Breeding and Acclimatization Institute—National Research Institute, Radzików, 05-870 Błonie, Poland; g.zurek@ihar.edu.pl; 8 Institute of Biology and Biotechnology, University of Rzeszów, Aleja Rejtana 16c, 35-959 Rzeszów, Poland; jaze28@interia.pl

**Keywords:** biomechanical tests, C_4_ plants, cell wall, cold tolerance, FTIR spectroscopy, *Miscanthus* × *giganteus*, photosynthetic activity, plasmodesmata

## Abstract

The cell wall plays a crucial role in plant growth and development, including in response to environmental factors, mainly through significant biochemical and biomechanical plasticity. The involvement of the cell wall in C_4_ plants’ response to cold is, however, still poorly understood. *Miscanthus* × *giganteus*, a perennial grass, is generally considered cold tolerant and, in contrast to other thermophilic species such as maize or sorgo, can maintain a relatively high level of photosynthesis efficiency at low ambient temperatures. This unusual response to chilling among C_4_ plants makes *Miscanthus* an interesting study object in cold acclimation mechanism research. Using the results obtained from employing a diverse range of techniques, including analysis of plasmodesmata ultrastructure by means of transmission electron microscopy (TEM), infrared spectroscopy (FTIR), and biomechanical tests coupled with photosynthetic parameters measurements, we present evidence for the implication of the cell wall in genotype-specific responses to cold in this species. The observed reduction in the assimilation rate and disturbance of chlorophyll fluorescence parameters in the susceptible M3 genotype under cold conditions were associated with changes in the ultrastructure of the plasmodesmata, i.e., a constriction of the cytoplasmic sleeve in the central region of the microchannel at the mesophyll–bundle sheath interface. Moreover, this cold susceptible genotype was characterized by enhanced tensile stiffness, strength of leaf wall material, and a less altered biochemical profile of the cell wall, revealed by FTIR spectroscopy, compared to cold tolerant genotypes. These changes indicate that a decline in photosynthetic activity may result from a decrease in leaf CO_2_ conductance due to the formation of more compact and thicker cell walls and that an enhanced tolerance to cold requires biochemical wall remodelling. Thus, the well-established trade-off between photosynthetic capacity and leaf biomechanics found across multiple species in ecological research may also be a relevant factor in *Miscanthus*’ tolerance to cold. In this paper, we demonstrate that *M. giganteus* genotypes showing a high degree of genetic similarity may respond differently to cold stress if exposed at earlier growing seasons to various temperature regimes, which has implications for the cell wall modifications patterns.

## 1. Introduction

The cell wall, a dynamic and complex structure, plays a crucial role in the control of plant growth, development, and response to the environment [1,2,3,4,5]. In particular, it is involved in the acclimation processes of plants exposed to various abiotic stresses, owing to the significant plasticity of the cell wall biochemical composition, molecular structure, and biophysical properties [3,6]. When exposed to cold, plants generally show multiple adaptive modifications in cell wall characteristics, which may affect wall polysaccharides, including cellulose, pectin, β-glucan [7,8,9,10,11], lignin [12,13], and phenolic compounds [14,15,16], as well as cell wall proteins [17,18,19]. However, we are still far from fully understanding cell wall involvement in the cold tolerance mechanisms of C_4_ plants.

*Miscanthus* × *giganteus*, a triploid interspecific hybrid between *Miscanthus sinensis* and *M. sacchariflorus*, a C_4_ grass of south-eastern Asian origin, is one of the most promising crops worldwide due to its high resource-use efficiency and biomass production, mainly for energy purposes. In contrast to other thermophilic species (maize, sorghum), *Miscanthus* is able to maintain a relatively high rate of CO_2_ assimilation at low ambient temperature; thus, it is considered as a reference of cold tolerance within C_4_ plants. 

*Miscanthus*’ cold tolerance allows it to achieve about 60% more productivity than maize [20], which can partly be related to the relatively early development of photosynthetically active leaves [21], as well as to the “balance” between a high rate of growth and stress tolerance [22]. The cold tolerance phenomenon of *Miscanthus* has been studied in several research projects and mainly concerns the analysis of physiological traits, including photosynthetic enzymes activity and the efficiency of photosystem II (PSII) [21,22,23,24,25,26,27,28,29,30,31]. In our earlier studies on the adaptation mechanisms of *Miscanthus* to low temperatures, the cold-induced modification of leaf physiology, anatomy, and some cell wall components, and changes in the expression of key enzymes in sugar metabolism and their products were also found [14,32]. Because the mechanism of C_4_ photosynthesis is characterized by the separation of its stages between two types of cells: mesophyll (primary CO_2_ binding) and bundle sheath cells (organic acid decarboxylation and acceptor restoration), the efficiency of this process depends on the effective intercellular transport of photosynthetic products. The transport of photoassimilates occurs in a symplasmic way, through micro-channels, i.e., plasmodesmata [33], and this type of transport is forced by the isolation of mesophyll from bundle sheath cells by a water-impermeable suberin layer [34]. In maize, a species closely related to *Miscanthus*, one of the mechanisms that determine a chilling sensitivity may be the changes in the ultrastructure of plasmodesmata in the photosynthetic pathway, as previously shown by our research team [35,36]. These changes have been related to the swelling of electron-dense substructures, so-called “sphincters”, and a decreased lumen of the cytoplasmic sleeve of plasmodesmata.

The role of the cell wall in plant adaptations to abiotic stresses has been analysed by not only using molecular, anatomical, physiological, and biochemical tools but also by means of biophysical methods, including vibrational spectroscopy and biomechanical tests. Particularly, FTIR was successfully used to monitor general changes in the biochemical profile of plants in response to stress [37], as well as to analyse cell wall plasticity in response to environmental stimuli [38]. Biomechanics is another useful methodology for researching plant response to an altered environment [37,39], particularly if the focus is on the cell wall [40]. The measurements of stiffness or strength may indirectly give insights into global changes in both the relative content of load-bearing wall macromolecules and the patterns of their intermolecular interactions [39]. Not only is this methodology fundamental in eliciting the role of cell walls in the control of growth [41,42,43], it also appears to be beneficial in the monitoring of cell wall plasticity in response to abiotic factors, which do not necessarily relate to growth rate [11,44,45,46,47,48].

It has been assumed that *M. giganteus*, originally imported from Asia and cultivated on a large scale in Europe, derives from the same genetic pool (cultivar “Hornum”) from Denmark [49]. Early molecular studies revealed the existence of several clones of *M. giganteus* in Europe [50], representing low genetic diversity [51,52]. Due to the origin of our plant material from the same genetic pool, a similar pattern of abiotic stress responses (here, to low temperature) in tested *Miscanthus* genotypes was expected. Nevertheless, some differences in the *Miscanthus* genotypes’ reaction to cold may occur, which may be connected with epigenetic changes that have appeared during somatic propagation or the distinct adaptability of specific genotypes to various microlocal environmental conditions.

The aim of this study was to verify the hypothesis that the differentiated physiological response to chilling among tested *M. giganteus* genotypes is associated with changes in the biomechanical and biochemical properties of the cell wall, as well as with the modification of the plasmodesmata ultrastructure, connecting cells in the photosynthetic pathway. For this purpose, we have analysed photosynthetic activity as well as cold-induced modifications of the plasmodesmata ultrastructure that may affect photoassimilate transport. Moreover, we have examined changes in the biochemical profile of the cell wall, using infrared spectroscopy, as well as the leaf/cell wall effective stiffness and strength in order to estimate the potential involvement of the cell wall in the control of the plants’ response to cold stress.

It is expected that this study will bring new insight into our understanding of cold-induced cell wall remodelling in C_4_ plants.

## 2. Materials and Methods

### 2.1. Plant Material and Growth Conditions

The rhizomes of *Miscanthus* × *giganteus* were derived from plantations located in three regions of Poland: M1—Bydgoszcz (53°17′ N, 18°04′ E), M2—Radzików, (52°21′ N, 20°64′ E), both plantations are the property of the Plant Breeding and Acclimatization Institute—National Research Institute (PBAI—NRI), and M3—Majdan Sieniawski (50°29′ N, 22°72′ E), a private plantation. Material selection for the study was based on meteorological data, where the rhizomes of three tested *Miscanthus* genotypes originated from locations with different values of monthly minimum and maximum temperatures. As a reference in genetic diversity analysis, two additional accessions, *Miscanthus sacchariflorus* (M4) and *Miscanthus sinensis* (M5) were used for DNA isolation. For the analysis of the cold effect, plants of three forms of *Miscanthus* (M1, M2, and M3) were grown in 5–litre pots, with a solid substrate, in a growth chamber with the following settings: 14/10 h light/darkness, irradiance 350 μmol quanta m^−2^ s^−1^ at temperature: 24/22 °C (day/night). In the phase of the fully developed third leaf and at the beginning of the light period, one half of the plants were transferred to a growth chamber with a low temperature: 12/10 °C (day/night) for 3 and 5 days (variants: 3d and 5d). The control (variants: c 3d and c 5d) were the plants not transferred to the cold. In each repetition of three independent experiments, at least 6 plants per variant were used.

### 2.2. Temperature Data

**Figure 1:**
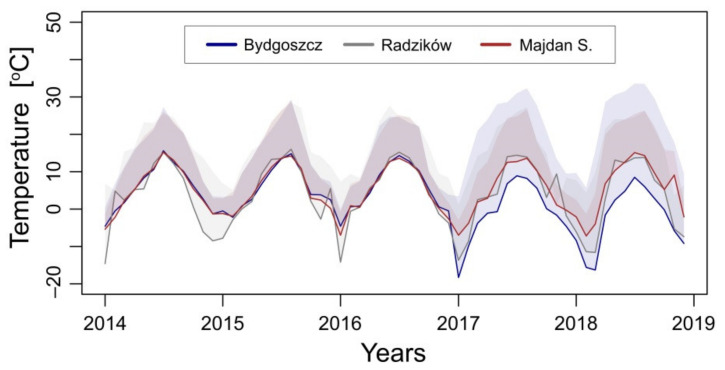
Monthly minimum and maximum temperatures for three localizations of *M. giganteus* plantations: Bydgoszcz (53°17′ N, 18°04′ E), Radzików (52°21′ N, 20°64′ E), and Majdan Sieniawski (50°29′ N, 22°72′ E), from which rhizomes were collected. Note the higher temperature values recorded in the location of Majdan Sieniawski (plantation with the M3 genotype rhizomes source) in contrast to the other two locations: Bydgoszcz (M1 genotype) and Radzików (M2 genotype), where relatively low values of monthly minimum temperatures were recorded. The data were obtained from meteorological stations owned by PBAI—NRI (Bydgoszcz, Radzików) and by IMWM—NRI (Majdan S.).

Temperature data were taken from meteorological stations located close to each plantation, i.e., in the Botanical Garden of the PBAI—NRI in Bydgoszcz and in the experimental fields of PBAI—NRI in Radzików (both meteorological stations are owned by PBAI—NRI), and in Tarnogród, about 8 km from Majdan Sieniawski (a station owned by Institute of Meteorology and Water Management—National Research Institute, Poland). The data included monthly maximum and minimum temperatures from five years (2014–2018), which are presented in Figure 1.

### 2.3. DNA Extraction and ISSR PCR

Total genomic DNA was isolated using hexadecyltrimethylammonium bromide (CTAB), according to Murray and Thompson (1980), with minor modifications. In brief, about 200–250 mg of fresh leaf tissue was collected from three forms of *M. giganteus* (M1, M2, and M3) and from *M. sacchariflorus* (M4) and *M. sinensis* (M5), as references. Tissue was placed in a 2 mL Eppendorf tube with 0.8 mL of freshly prepared CTAB solution and ß-mercaptoethanol, and 3 stainless steel balls of 4 mm diameter were added to the extract. The samples were homogenized in a mixer mill (MM301, Retsch, Haan, Germany) at frequency 3000/min for 5 min. Then, the samples were carefully vortexed and incubated in a TS thermomixer (BioSan Laboratories Inc, Thermo-Shaker SC-24C, Warren, MI, USA) heating block with intensive shaking for 35–40 min at 60 °C. The next stages were carried out according to Murray and Thompson [53]. The concentration and purity of DNA were determined using a spectrophotometer (NanoDrop 3300, Thermo Fisher Scientific, Waltham, MA, USA). Polymerase chain reactions (PCRs) were performed in low-profile PCR tubes strips (Bio-Rad Laboratories, Hercules, CA, USA) in a Mastercycler thermocycler (Merck KGaA, Darmstadt, Germany). The reactions were carried out under the following conditions: total reaction volume of 25 μL, 25 ng of template DNA, 1× PCR buffer (MBI Fermentas, Waltham, MA, USA), 2.5 mM MgCl_2_, 0.2 mM dNTP, 1 U of Taq DNA polymerase (MBI Fermentas, Waltham, MA, USA), and 1 µM of each ISSR primer (Merck KGaA, Darmstadt, Germany). PCR reactions were performed within the following parameters: initial denaturation at 94 °C for 3 min, 40 cycles at 94 °C for 1 min, 48–63 °C for 1 min, and 72 °C for 1 min, followed by a final extension at 72 °C for 5 min. Based on studies of Cichorz et al. [49], 15 primers were primarily used, out of which 13 generated stable and clear bands and were used for further tests. The PCR products were electrophoresed on a 1.7% agarose gel (Bio Standard, Prona Agarose, Burgos, Spain) in 1× TBE buffer for 2.5 h. Amplification products were visualised under UV light after ethidium bromide staining. The images of the gels were made using a Gel Logic 200 system (Eastman Kodak Company, New York, NY, USA). A binary genotyping matrix with 0–1 scores was used to calculate a pair-wise genetic similarity matrix on the basis of Jaccard’s coefficient [54] using the R package distantia [55], and then a cluster analysis was performed to construct an unweighted pair group method using arithmetic mean (UPGMA) dendrogram.

### 2.4. Photosynthetic Parameters Measurements

An LI-6400XT gas-exchange system and chamber, with an RGB Light Source (LI-COR 6400–18, LI-COR, Lincoln, NE, USA), was used for the determination of the following parameters: net CO_2_ assimilation, the quantum yield of photosystem II (ϕ_PSII_), and maximal photochemical efficiency of photosystem II (*F*_v_/*F*_m_). Measurements were performed at 500 μmol (photon) m^−2^ s^−1^ of the photosynthetically active radiation (PAR) range with a leaf temperature of 25 °C under the following ambient conditions: 390 ± 5 μmol mol^−1^ CO_2_ and 210 mmol mol^−1^ O_2_. For *F*_v_/*F*_m_ determination, plants were dark-adapted for 30 min at 24 °C. At least ten plants per experimental variant were used in each of the three independent experiments.

### 2.5. Tensile Mechanical Tests

Leaf tissue and cell wall mechanical properties were determined for fully hydrated and frozen-thawed (−20 °C) rehydrated samples, using tensile tests (Instron, model 5542, London, UK). At least twenty samples (3 × 30 mm) per genotype and treatment were collected from the region located between the upper one-third and the middle part of a fully developed third leaf. Full hydration of samples was obtained as a result of keeping them between wet paper towels for 24 h at 5 °C. The specimens were mounted, at one end, by the grips to the load cell (10 N capacity) and, at the other end, to the immovable part of the instrument at a gauge length of 10 mm. Strips of fine grit sandpaper were used to prevent slippage of the sample from the grips during the measurements. A load vs. displacement relationship was determined at the extension rate of 1 mm/min and was explored for calculating stiffness (Young’s modulus) and strength. Stiffness was calculated as the ratio of stress (load per sample cross-section) and the relative extension at the initial linear region of the recorded curve. The tensile strength was defined as the ratio of the breaking load to the sample’s cross-sectional area. The data were collected and processed by means of Instron’s Bluehill 2 software. The thickness of the samples was determined using a dial gauge at an accuracy of 0.01 mm (mean of three locations). Only samples that showed no signs of clamping-related failure were considered for further calculations.

### 2.6. Infrared Spectroscopy

Mid-infrared spectroscopy was used to detect genotype-related responses to chilling in the biochemical profile of the cell wall. The attenuated total reflectance Fourier transform infrared (ATR—FTIR) method was applied. The spectral analysis was performed for eight to ten samples per genotype and treatment. Lyophilized and homogenized into powder (ball mill, MM 400, Retsch, Haan, Germany), the samples were deposited onto the one-bounce diamond crystal of an ATR accessory (Smart Orbit, Thermo Scientific, Madison, WI, USA) coupled to the iZ10 module of a Nicolet iN10 MX spectrometer (Thermo Fisher Scientific, Waltham, MA, USA), equipped with a deuterated triglycine sulphate (DTGS) detector and a KBr beam splitter. Two hundred and fifty-six spectra were collected and co-added within the wavelength range between 4000 and 400 cm^−1^ at 4 cm^−1^ resolution. The ATR diamond crystal was cleaned carefully before successive measurements, and the possible presence of analyte residues on the crystal surfaces was examined by recording the residual spectra after cleaning. The spectra were recorded, averaged and baseline corrected using OMNIC software (v. 9.0, Thermo Fischer Scientific Inc.). The data were normalized to the unit area within the 1800–900 cm^−1^ wavenumber region and further explored using ChemoSpec [56] package in R programming language [57].

### 2.7. Ultrastructure of Plasmodesmata

For the analysis of the plasmodesmata ultrastructure, the material was prepared according to Bilska and Sowiński [35], with minor modifications. Briefly, samples (1 × 2 mm) were taken from the region located between the upper one-third and the middle part of the fully developed third leaf. The material was immediately fixed in 4% glutaraldehyde (with 0.5% tannic acid) in 0.1 M phosphate buffer, pH 7.3, for 4 h and was post-fixed with 1% osmium tetroxide for 2 h (both at 4 °C). After dehydration (ethanol 10–100%), the samples were embedded in Agar 100 resin (Agar Scientific Ltd, Stansted, UK) and polymerized for 24 h at 60 °C. Ultrathin (80 nm) sections were cut with a diamond knife on a Reichert Ultracut (Wien, Austria) ultramicrotome in the Laboratory of Microscopic Techniques, Faculty of Biological Sciences (University of Wrocław, Poland). Stained sections (with uranyl acetate and lead citrate) were examined under a transmission electron microscope (model JEM 1400; JEOL Ltd., Tokyo, Japan), equipped with a CCD camera (MORADA G2, EMSIS GmbH, Münster, Germany). 

The cross-sectional area of plasmodesmata (Pd) at mesophyll–mesophyll (MS–MS) and mesophyll–bundle sheath (MS–BS) interfaces were evaluated from the electron microscope photographs (taken under ×60,000 and ×100,000 primary magnifications) which were produced from at least 6 specimens (per genotype and experimental variant). The measurements were performed using iTEM software (Olympus Soft Imaging Solution, Münster, Germany) for thirty individual Pd (from each interface). Only clearly visible and showing undisturbed shape Pd were considered.

### 2.8. Statistical Analysis

Statistics applied for data from the experiments were conducted in a completely random design and were performed in R [57]. The data (except cross-sectional plasmodesmata area) were initially subjected to factorial analysis (a three-way ANOVA model) using the R base package [57], and the model output was used as the argument for the emmeans function from the emmeans package [58] to calculate estimated marginal means (EMMs). The genotype, treatment (control-chilling), and stress duration (3 and 5 days) constituted factors in the model. Six planned contrasts were created for the factor combinations, and the difference between the EMMs were used to assess the cold treatment effect for each of the three genotypes, at particular levels of cold duration. The “holm” *p*-value adjustments were applied to control Type I error inflation as a result of multiple testing [58]. The cross-sectional areas of plasmodesmata were analysed using two-way ANOVA and post-hoc Tukey’s Honestly Significant Difference (HSD) test using an R base package [57].

## 3. Results

### 3.1. Genetic Similarity

**Figure 2:**
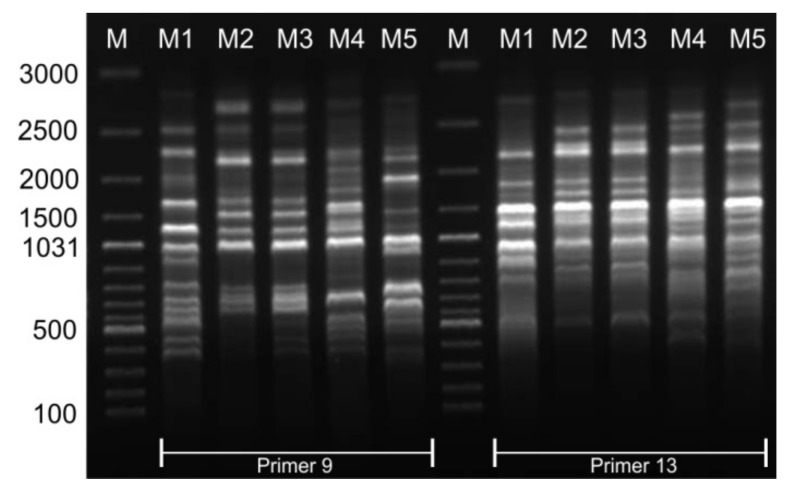
Electrophoresis banding patterns of PCR amplification products using ISSR primers 9 and 13 for five tested *Miscanthus* genotypes. Lane abbreviations: M, 100 bp DNA ladder (MBI Fermentas, Waltham, MA USA); M1, *M. giganteus* “Bydgoszcz”; M2, *M. giganteus* “Radzików”; M3, *M. giganteus* “Majdan Sieniawski”; M4, *M. sacchariflorus*; and M5, *M. sinensis*.

Thirteen ISSR primers were finally used in the analysis of the genetic similarity of the tested *Miscanthus* genotypes, which generated a total of 314 bands, of which 71% were polymorphic. The number of bands obtained for a single primer was in the range of 9–39, and the approximate size of the amplified products was from about 390 to 2700 bp. The examples of gels with product amplification using primers 9 and 13 are given in Figure 2.

**Figure 3:**
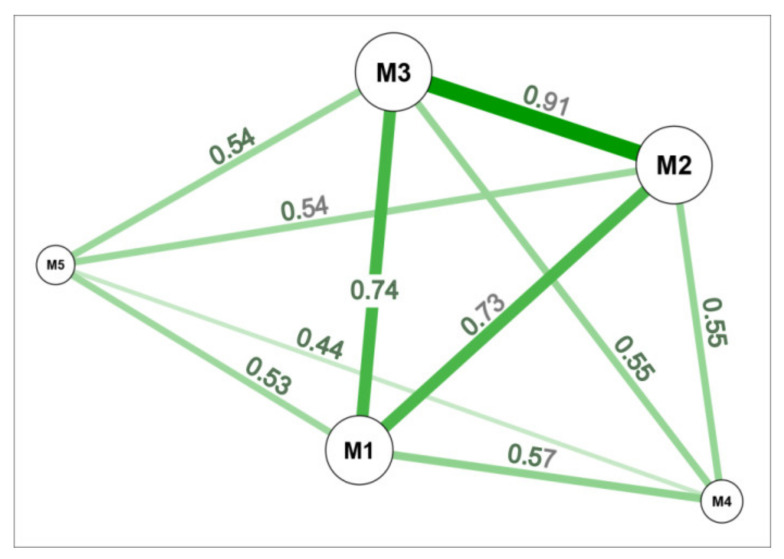
Genetic similarity between five genotypes of *Miscanthus*: M1, *M. giganteus* “Bydgoszcz”; M2, *M. giganteus* “Radzików”; M3, *M. giganteus* “Majdan Sieniawski”; M4, *M. sacchariflorus*; and M5, *M. sinensis*. The plot was constructed on the basis of ISSR data using the R package distantia package [59] and the Jaccard similarity index [54].

High genetic similarity was observed within three *M. giganteus* genotypes (M1, M2, M3) where the highest coefficient (0.91) was noted between the M2 and M3 genotypes, while the lower coefficients (0.73 and 0.74) were found for the M1-M2 and M1-M3 pairs, respectively (Figure 3). A relatively wide diversity with similarity coefficient in the range of 0.53–0.57 was observed between *M. giganteus* (M1) and both *M. sacchariflorus* (M4) and *M. sinensis* (M5). The lowest genetic similarity coefficient (0.44) was observed for pair M4 and M5.

### 3.2. Photosynthetic Activity

**Figure 4:**
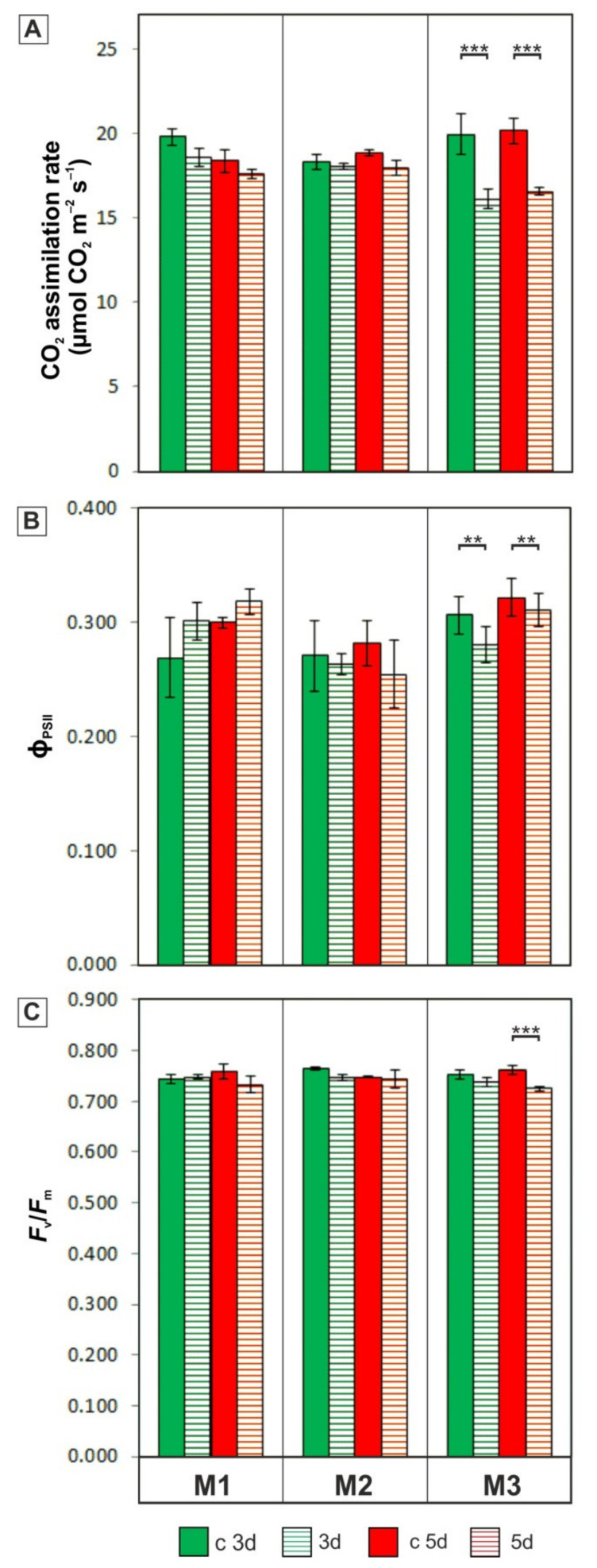
Net CO_2_ assimilation (**A**), the quantum yield of photosystem II, ϕ_PSII_ (**B**), and maximal photochemical efficiency of photosystem II; *F*_v_/*F*_m_ (**C**) in the control (c 3d and c 5d) and chilled for 3 and 5 days (3d and 5d) for plants of three *M. giganteus* genotypes (M1, M2, and M3). Bars represent means ± SD; asterisks indicate a significant effect of chilling based on the planned contrasts for EMMs derived from a three-way ANOVA model. The “holm” adjustment was applied to control the multiple comparison. ** *p* ≤ 0.01, *** *p* ≤ 0.001. Data were collected from at least 10 plants in each of the three independent experiments.

Both variants of cold stress (3d and 5d) caused a significant reduction of photosynthesis (net CO_2_ assimilation) in leaves of the M3 plants to a value of about 16 μmol CO_2_ m^−2^ s^−1^, whereas photosynthesis in the control plants was in the range of 18–20 μmol CO_2_ m^−2^ s^−1^ for M1 and M2 and about 20 μmol CO_2_ m^−2^ s^−1^ for the M3 genotype (Figure 4A). Similarly, the quantum yield of photosystem II (ϕ_PSII_) clearly decreased in the chilled plants of the M3 genotype, while in M1 and M2, these changes were not observed (Figure 4B). The maximal photochemical efficiency of PSII (*F*_v_/*F*_m_) for the control plants of the tested genotypes was in the range of 0.74–0.76 (Figure 4C). A significant decrease in *F*_v_/*F*_m_ was observed after a prolonged period of chilling treatment (5d) in the leaves of M3 plants.

### 3.3. Biomechanical Tests

**Figure 5:**
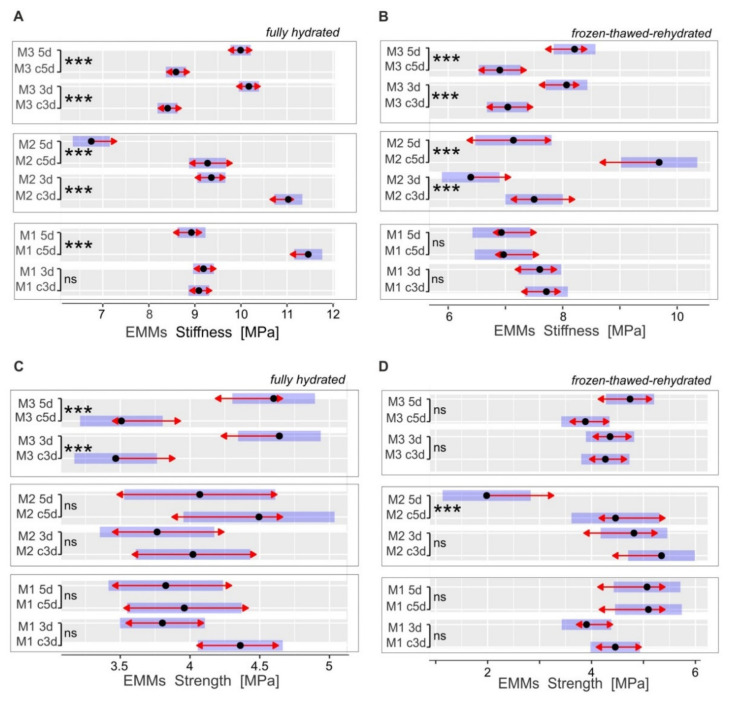
Tensile mechanical parameters of leaves for control (c3d and c5d) and cold-treated (3d and 5d) plants of three *Miscanthus* genotypes (M1, M2, M3). Stiffness (**A**,**B**) and strength (**C**,**D**) were determined for fully hydrated (**A**,**C**) and frozen–thawed–rehydrated (**B**,**D**) samples (*n* = 18–24). The estimated marginal means EMMs (black dots) were derived using a three-way ANOVA model. Horizontal blue bars show 95% confidence in the means. The comparison arrows (red) are added to visualize the homogeneity of groups in the planned contrast test (the overlap of the lines indicates a lack of significant difference). The “holm” adjustment was applied to control the comparison multiplicity. *** *p* ≤ 0.001, ns—*p* > 0.05.

Unidirectional tensile tests were performed using a universal testing machine (Instron) to determine changes in the mechanical parameters of leaf tissue (fully hydrated samples) and the cell wall (frozen-thawed-rehydrated preparations) [60], associated with the cold response in the tested *Miscanthus* genotypes (Figure 5). The latter approach removed the turgor effect, which allowed for the mechanical properties of the leaf sample to be exclusively attributed to the cell wall material. Considering the large structural heterogeneity of leaves at the growth stage, the examined parameters provided values of the “effective” stiffness and strength, which take into account both, the variability in the mechanical properties of leaf tissues and the cell wall, as well as the relative contributions of various tissues in the load-bearing capacity of the specimens [39]. The presence of turgor generally elevates the stiffness of leaf samples; however, in reference to the strength of the samples, the turgor effect was not clear, indicating much more complex phenomena.

Cold treatment (variants 3d and 5d) led to an increase in stiffness (Figure 5A,B) and strength (Figure 5C) in the M3 genotype, while the M1 and M2 genotypes generally showed opposite responses. Namely, the tensile stiffness increased by about 20% in fully hydrated samples (3d and 5d) and by 14% (3d) and 20% (5d) in frozen-thawed-rehydrated samples of the M3 genotype. In the M2 genotype, fully hydrated and frozen-thawed-rehydrated samples showed a decrease in tensile stiffness by 12–16% (3d) and by 24–28% (5d). Finally, the M3 genotype was characterized by about a 30% increase in tensile strength in fully hydrated samples, in both variants of chilling treatment (3d and 5d). A decrease in this parameter (−36%) was observed in the frozen–thawed–rehydrated leaves of the M2 genotype, after 5 d of chilling treatment (Figure 5D). Overall, we observed changes in both the tissue and the cell wall mechanical properties in response to chilling, to various extents, depending on the genotype examined.

### 3.4. Infrared Spectroscopy

**Figure 6:**
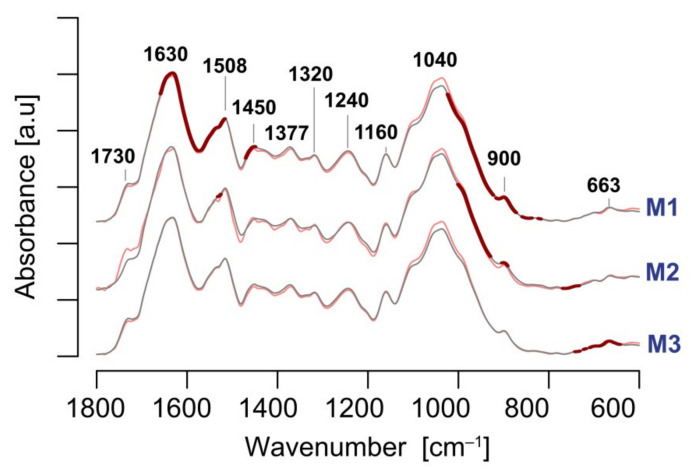
Mean infrared spectra of leaf cell wall material collected from the control (grey) and chilled (red) plants for three *M. giganteus* genotypes: M1 (“Bydgoszcz”), M2 (“Radzików”), and M3 (“Majdan Sieniawski”). Spectral regions indicating significant differences between the means (control vs. chilled plants) are depicted by thick red lines. The significance (*p* < 0.05) of the treatment was estimated using the planned contrasts after the three-way ANOVA and were calculated for each wavenumber within the 1800–600 cm^−1^ region (*n* = 8–10). The “holm” correction was used for the multiplicity adjustment.

We analysed the mean spectra of the cell wall material extracted from leaves of the control and chilled plants. The most prominent bands are distinguished by added wavenumbers (Figure 6). All spectra were generally similar for all genotypes, showing the most pronounced peaks at around 1630, 1508, 1240, and 1040 cm^−1^. The presence of a relatively strong peak at around 1508 cm^−1^, assigned to aromatic skeletal vibrations [61,62,63], indicates extended lignification of the cell wall in the collected leaf samples. Other bands at 1465 and 1377 cm^−1^, related to the C-H vibrations of the CH_2_ and CH_3_ functional groups, may be related to lipids, proteins and lignin compound. The peaks at 1160 and around 1040 cm^−1^, attributed to the C-O vibrations, usually coupled with the C-C vibrations, reflect mainly the presence of cellulose [64,65,66].

We did not detect large visible changes in the general spectral pattern as a result of chilling. Statistics based on the planned contrasts for the EMMS of absorbance, calculated based on the three-way ANOVA model, separately for each wavenumber, showed that some regions of spectra were significantly affected by chilling (highlighted by means of a thick red line in Figure 6). The differences were generally higher at the third day of stress versus two days later, which indicated a transient impact of chilling on the biochemical profile of the cell wall (Supplementary Figure S1). The changes are also genotype dependent. The M1 genotype showed the most prominent changes in the spectra, mainly in the region between 1700–1500 cm^−1^ and the region covering the shoulder of the polysaccharide band, centred at about 1040 cm^−1^, whereas the M2 genotype showed alterations only at the shoulder of the polysaccharide band. Finally, M3 plants displayed a response in the region close to 700–600 cm^−1^, which is consistent with our earlier report [32]. Visual inspection of the mean spectra in the region between 1150 and 1000 cm^−1^ revealed a slight increase of global polysaccharide content due to the cold, at the expense of non-carbohydrate compounds of the cell wall.

A deep insight into the details of the biochemical changes of the cell wall in response to cold seems difficult since the spectral regions related to cold response may be assigned to multiple cell wall compounds, particularly, to highly coupled vibrational modes of polysaccharide backbones. Nevertheless, we can infer that spectral features related to cellulose and non-cellulosic polysaccharides were not significantly affected by chilling in these experiments (similar shape of the carbohydrate band of 1150–1000 cm^−1^). 

### 3.5. Plasmodesmata Ultrastructure

**Figure 7:**
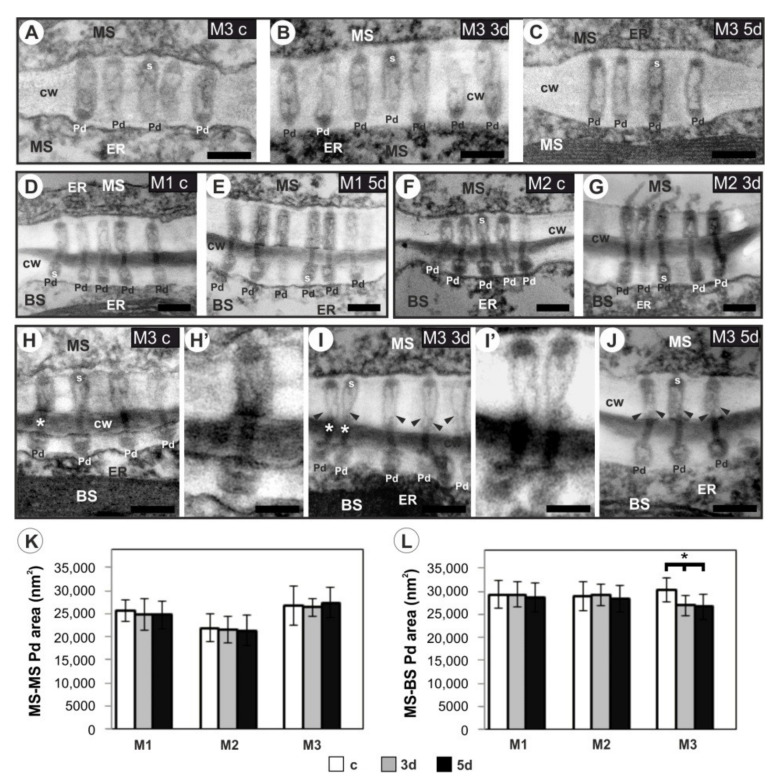
Examples of typical electron microscope images of plasmodesmata (**A**–**J**) and the results of the plasmodesmata area analysis (**K**,**L**) at the interface of mesophyll–mesophyll, MS–MS (**A**–**C**, **K**) and mesophyll–bundle sheath cells, MS–BS (**D**–**J**,**L**). The control, c (non-chilled) plants (**A**,**D**,**F**,**H**,**H’**), chilled plants for 3 days, 3d (**B**,**G**,**I**,**I’**) and for 5 days, 5d (**C**,**E**,**J**). Note electron-dense elements of the sphincters (s) in the neck regions on both sides of the plasmodesmata. Arrowheads indicate the clear constriction of the cytoplasmic sleeve in the central region of the MS–BS plasmodesmata in the leaves of the M3 genotype (**I**,**J**). Plasmodesmata marked with asterisks (**H**,**I**) are at higher magnification (**H’**,**I’**) for better visualization of the ultrastructure. Abbreviations: M1, *M. giganteus* “Bydgoszcz”; M2, *M. giganteus* “Radzików”; M3, *M. giganteus* “Majdan Sieniawski”; Pd, plasmodesmata; CW, cell wall; ER, endoplasmic reticulum; s, sphincter; Scale bar = 200 nm (**A**–**J**); = 100 nm (**H’**,**I’**). The Pd area (**K**,**L**) was measured from thirty individual Pd at the MS–MS or MS–BS interface for at least six plants and three independent experiments. Bars represent the means ± SD. *—significant effect of chilling (Tukey’s HSD test, *p* < 0.001).

In the control leaves of all three tested *Miscanthus* genotypes, the plasmodesmata at the interfaces of mesophyll–mesophyll (MS–MS) and mesophyll–bundle sheath cells (MS–BS) were arranged in groups called “pit fields” and represented the structure characteristic of C_4_ grasses [67,68]. Namely, an electron-dense internal substructure called the “sphincter” [68], commonly found in other C_4_ species [69], was observed at both sides of the plasmodesmata at MS–MS and MS–BS interfaces (e.g., Figure 7A,D,H). Microscopic analysis revealed no significant changes in the plasmodesmata ultrastructure between MS cells in all *Miscanthus* genotypes tested (Figure 7A–C), whereas the cold-treatment (both variants: 3d and 5d) led to the constriction of the cytoplasmic sleeve of plasmodesmata between MS and BS cells in the leaves of M3 genotype (Figure 7I–J). Such modifications of this type of plasmodesmata have not been observed in the leaves of M1 and M2 genotypes (Figure 7D–G).

The analysis of the cross-sectional area of the plasmodesmata on the basis of microscopic observations showed a lack of differences between MS cells in the three *Miscanthus* genotypes and at the MS–BS interface in the M1 and M2 genotypes (Figure 7K–L). In turn, both variants of cold (3d and 5d) caused a reduction in the area of MS–BS Pd compared to the control plants in the leaves of the M3 genotype (*p* < 0.001). 

## 4. Discussion

This study investigates possible mechanisms underlying differential cold responses of *M. giganteus* genotypes with a focus on the plasmodesmata ultrastructure and the role of the cell wall in relation to its mechanics and biochemical remodelling.

### 4.1. (Micro)Climatic Conditions of Plantations versus the Adaptability of Photosynthetic Activity of M. giganteus Genotypes to Low Temperature

The rhizomes used in this study originated from three localizations in Poland with different climatic conditions, monitored by temperature. Meteorological data from a 5-year period showed the lowest values of the minimum temperature for the locations Bydgoszcz and Radzików, and the highest values for Majdan Sieniawski (Figure 1). The analysis of genetic variation detected high genetic similarity coefficients within the three *Miscanthus* genotypes tested (Figure 3). Similarly, a lack of or low genetic variation was detected within three *M. giganteus* accessions (“Canada”, “Germany”, “Great Britain”) from a collection at PBAI—NRI [49] and within 11 accessions from collections at RGB Kew and ADAS Arthur Rickwood Research Station, Cambridgeshire, UK [70]. In the comparative studies of genetic variation of fifty North American and European *M. giganteus* accessions, “critical” low genetic diversity was observed, whereas only some variation was noted for eight new triploid *M. giganteus* genotypes [71]. Low genetic diversity limits the geographical range of individual *Miscanthus* genotypes and the potential for adaptation to various environmental conditions, especially in the case of newly established plantations [51,72]. However, the introduction of new, fertile *Miscanthus* germplasm from Asia and its transfer to Europe helped to create new genetically diverse accessions with improved yield under different climatic conditions [73].

The physiological response to stress is most often visualized by the rate of gas exchange and chlorophyll fluorescence parameters, which can be a useful tool to estimate the tolerance level of plants to various environmental factors. This approach has been used in the rapid assessment of drought tolerance of the field-grown wheat varieties [74], the analysis of dynamics of salinity stress in *A. thaliana* seedlings [75], and in the search for adaptation mechanisms to the cold springs in maize [76] as well as in the comparative analysis of the cold tolerance in two *Miscanthus* genotypes [30]. In our experiments, chilling treatment (10–12 °C) did not cause changes in net CO_2_ assimilation (Figure 4A), the quantum yield of photosystem II (ϕ_PSII_) (Figure 4B), or maximal photochemical efficiency of photosystem II (*F*_v_/*F*_m_) (Figure 4C) in the M1 and M2 genotypes. The maintenance of high photosynthesis efficiency in the cold may be the result of adaptive mechanisms acquired in these genotypes during field growth at the relatively low temperatures occurring at these two localizations (Figure 1), in which repetitive patterns of DNA methylation responsible for the regulation of cold-responsive genes may be involved. The rapid epigenetic response to cold has been demonstrated for many plant species, including maize [77], rye [78], and *Arabidopsis* [79], and the possibility of methylation pattern inheritance [80] may have repercussions in elucidating adaptation mechanisms to low temperature [81]. Probably, in our case, “cold shock” conditions in two localizations, occurring especially during early spring, were conducive to the formation of defence mechanisms, leading to the acclimatization of the M1 and M2 genotypes to low temperatures. Such mechanisms can be related to the high activity of key enzymes responsible for carbon fixation, i.e., pyruvate orthophosphate dikinase (PPDK) [27,29] and Rubisco [28], and/or with avoiding photo-damage by activating alternative pathways to neutralize absorbed excitation energy [24]. It has also been found that priming, including a 20-day cold-treatment at 10 °C, at the early stage of *M. giganteus* plants’ development has a positive effect on the photosynthetic activity (e.g., through increased chlorophyll production) in the subsequent stages of plant growth under unfavourable environmental conditions [13]. This may be beneficial for the expansion of *Miscanthus* cultivation to areas in colder European climates through breeding promising new hybrids developed using advanced methods of molecular selection [73,82]. 

### 4.2. Are Cold-Induced Disturbances in Photosynthetic Activity Related to the Plasmodesmata Ultrastructure?

In the M3 genotype, a significant reduction of CO_2_ assimilation and ϕ_PSII_ after both periods (3d and 5d) of chilling treatment were noted (Figure 4A,B). Moreover, in this genotype, the maximal photochemical efficiency of PSII (*F*_v_/*F*_m_) was significantly lower in plants chilled for 5 days compared to the control (Figure 4C). Such changes in this genotype have not been observed in our earlier studies on the comparative cold response of *Miscanthus* and maize plants [14]. In that work, *M. giganteus* was used as a kind of reference for maize, which is generally considered to be a cold-sensitive species among C_4_ plants, and the detection of changes in photosynthetic activity was possible for *M. giganteus* in this experiment, probably due to the use of a higher temperature, in the range of 12–14 °C (where, in the present work, it was: 10–12 °C). Similarly, a clear decrease in CO_2_ assimilation was observed for several genotypes of *M. giganteus* after 1 day of exposure to cold temperatures of 10 °C [21].

The cold-induced changes in photosynthesis were accompanied by modifications of the ultrastructure of transport microchannels—the marked constriction of the cytoplasmic sleeve in the central region of the plasmodesmata, between mesophyll and bundle sheath cells in the chilled leaves of the M3 genotype (Figure 7I–J). In this genotype, the MS–BS plasmodesmata area was significantly lower in chilled plants compared to the control (Figure 7L). In *Miscanthus* and other C_4_ plants, the plasmodesmata are of particular importance because their permeability determines the efficient transport of assimilates between different types of photosynthetically active cells. Earlier, it has been demonstrated that carbon transfer between the C_4_ and the C_3_ cycle is slowed down at low temperatures [83], and the changes in the kinetics of transport of photosynthetic products may result in the overaccumulation of sucrose and/or starch [84], leading to general disturbances in sugar metabolism [32]. Ultrastructural modifications of plasmodesmata, similar to those observed in the M3 genotype, were found in the chilling-sensitive maize line, in other types of plasmodesmata between bundle sheath, and vascular parenchyma cells [35]. In that study, the strong inhibition of photosynthesis was also noted in this maize line, and calreticulin was indicated as a regulatory factor of plasmodesmata permeability due to the activity of this protein in the calcium buffering in plant cells [85]. The activity of calcium-dependent proteins temporarily adjusted to low temperature was found in the study of the response to freezing in *Fritillaria imperialis* [86]. In our study, it can be assumed that the decrease in the lumen of the cytoplasmic sleeve of plasmodesmata in the leaves of M3 plants (Figure 7I–J,L) may be responsible for the inhibition of CO_2_ assimilation in this genotype (Figure 4A). 

### 4.3. The Possible Relationship between Photosynthetic Response to Stress and the Leaf Biomechanics

Recently, some studies [87,88] highlighted links between the photosynthetic activity of leaves and leaf biomechanics. Accordingly, these reports examined tree species with higher leaf mechanical strength, showing generally reduced photosynthetic efficiency. There was an attempt to explain this as (i) a trade-off between the allocation of nitrogen into the cell wall and in the photosynthetic proteins, and/or (ii) the reduction of gas diffusion conductance associated with wall thickening and/or the more compact structure of the walls [88,89].

Our studies showed that leaf biomechanical properties (Figure 5) are related to the response to chilling manifested through photosynthetic activity. The M3 genotype showed improvement in leaf strength and stiffness, along with a reduction of net CO_2_ assimilation compared to other genotypes. The increase in stiffness in frozen–thawed–rehydrated preparations seems to be particularly interesting in this respect. This parameter provides information on the contribution of the cell wall itself to leaf biomechanics. The improvement in stiffness due to cold treatment may be attributable to an increase in the amount of cell wall material, altered wall composition, and altered intermolecular links. Considering the FTIR measurements, there is no evidence for significant modification of the wall chemical composition in M3 plants due to cold, which suggests that cell wall thickening is primarily responsible for leaf stiffness. This is in accordance with our previous studies, where this genotype response to cold was investigated using electron microscopy [14]. Therefore, the increase in stiffness (a parameter per unit cross-sectional area of the leaf samples) of M3 due to chilling reflects a leaf structure that is more compactly filled with cell wall material. Such biomechanical strengthening of leaves may affect gas conductance to mesophyll tissues and thus the rate of gas exchange between the leaf and the environment [89].

Genotypes more tolerant to cold (M1 and M2) showed the opposite effect or a lack of change in tissue/wall mechanics that allowed them to maintain efficient photosynthesis under stress. These data may be considered as an indication that the photosynthesis–leaf biomechanics relationship, reported also for a set of species in ecological studies [88,89], may be relevant for understanding plant response to chilling.

The present data also suggest that cell wall mechanics may have an effect on the plasmodesmata ultrastructure, as genotypes characterized by more rigid cell walls showed relatively less expanded plasmodesmata. However, this requires more extended studies. 

### 4.4. Cold-Induced Alteration in Biochemical Profile of the Cell Wall Is Temporary and Genotype Dependent

Infrared spectroscopy is a convenient high-throughput tool for the inspection of general changes in the biochemical profile of the cell wall and has been successfully applied for cell wall mutant identification and in searching for genetic variability, developmental modifications, and plant response to an altered environment [64,90,91,92,93,94,95]. The technique has also been applied to characterize *Miscanthus* plant organs and their biomass compounds [91,96,97,98,99,100,101].

We used this technique to evaluate whether (i) cold treatment altered general cell wall biochemistry, and (ii) the response is genotype-dependent. All genotypes showed altered infrared spectra as a result of cold stress, but the response was temporary since the difference between the control and the treated plants after 5 days was much lower than after the 3-day cold treatment (Supplementary Figure S1). The genotypes also showed different mean infrared spectra which were affected by the cold stress. The most pronounced impact of cold was observed for the M1 genotype, while M3 plants were only minimally impacted.

The changes concerned a wall matrix, as spectral bands related to cellulose were not significantly affected, based on the spectra inspection within the carbohydrate region. In addition, an increase of absorbance was observed for the carbohydrate region in all genotypes, which indicates increased investment of assimilates for the synthesis of wall carbohydrates at the expense of non-carbohydrate compounds, resulting from plant exposition to chilling. This supports, to some extent, the thesis discussed above, which assumed a trade-off between CO_2_ exchange rate and leaf biomechanical strength.

## 5. Conclusions

This work showed that the three genotypes of *Miscanthus* × *giganteus*, despite high genetic similarity, may differ in the cold stress tolerance if they were previously exposed to various temperature regimes. The observed reduction in the assimilation rate and disturbance of chlorophyll fluorescence parameters in the M3 genotype under cold conditions were most likely influenced by changes in the ultrastructure of the plasmodesmata, i.e., constriction of the cytoplasmic sleeve in the central region of the microchannel at the mesophyll–bundle sheath interface. The maintenance of relatively high photosynthetic activity in M1 and M2 genotypes may be due to their ability to acclimatize to the low temperature that was acquired in field during their growth and development. The most susceptible to chilling was M3 genotype, which showed specific responses in terms of leaf biomechanics compared to genotypes that maintained high photosynthetic activity under cold stress, and the slightest changes in the biochemical profile of the cell wall as revealed by infrared spectroscopy. This suggests that the plasticity of the cell wall induced by low-temperature stress may support these acclimatization processes. This study also indicates that the trade-off between photosynthesis capacity and leaf biomechanics, reported earlier in an ecological context, may drive, to some extent, the mechanism of cold tolerance in *Miscanthus*.

## Data Availability

No data being reported.

## Supplementary Materials

The following are available online at https://www.mdpi.com/article/10.3390/cells11030547/s1, Figure S1: Mean infrared spectra collected from cell wall material for the control (grey) and chilled (red) plants of three *Miscanthus* genotypes.
