# Supplementary material for: Cell Wall Properties Determine Genotype-Specific Response to Cold in Miscanthus × giganteus Plants
Source: Cells. 2022 Feb 4;11(3):547. doi: 10.3390/cells11030547 (PMC8834381; doi:10.3390/cells11030547)
Supplement: Supplementary file 1 [file cells-11-00547-s001.zip › cells-1472818-supplementary.pdf]

cells MDPI

Supplementary Material

Cell Wall Properties Determine Genotype-Specific Response to Cold in *Miscanthus* × *giganteus* Plants

Anna Bilska-Kos [1,*], Aleksandra Pietrusińska [2], Szymon Suski [3], Agnieszka Niedziela [1], Anna M. Linkiewicz [4,5], Włodzimierz Majtkowski [6], Grzegorz Żurek [7] and Jacek Zebrowski [8]

1 Department of Biochemistry and Biotechnology, Plant Breeding and Acclimatization Institute—National Research Institute, Radzików, 05-870 Błonie, Poland; a.niedziela@ihar.edu.pl
2 National Centre for Plant Genetic Resources, Plant Breeding and Acclimatization Institute—National Research Institute, Radzików, 05-870 Błonie, Poland; a.pietrusinska@ihar.edu.pl
3 Laboratory of Electron Microscopy, Nencki Institute of Experimental Biology of Polish Academy of Sciences, 3 Pasteur, 02-093 Warsaw, Poland; soosky@o2.pl
4 Molecular Biology and Genetics Department, Institute of Biological Sciences, Faculty of Biology and Environmental Sciences, Cardinal Stefan Wyszyński University, Wóycickiego 1/3, 01-938 Warsaw, Poland; a.linkiewicz@uksw.edu.pl
5 Genetically Modified Organisms Controlling Laboratory, Plant Breeding and Acclimatization Institute—National Research Institute, Radzików, 05-870 Błonie, Poland
6 Botanical Garden, National Centre for Plant Genetic Resources, Plant Breeding and Acclimatization Institute—National Research Institute, Jeździecka 5, 85-867 Bydgoszcz, Poland; w.majtkowski@ihar.edu.pl
7 Department of Bioenergetics, Quality Analysis and Seed Science, Plant Breeding and Acclimatization Institute—National Research Institute, Radzików, 05-870 Błonie, Poland; g.zurek@ihar.edu.pl
8 Institute of Biology and Biotechnology, University of Rzeszow, Aleja Rejtana 16c, 35-959 Rzeszow, Poland; jaze28@interia.pl
* Correspondence: a.bilska@ihar.edu.pl; Tel.: +48-22-733-45-41

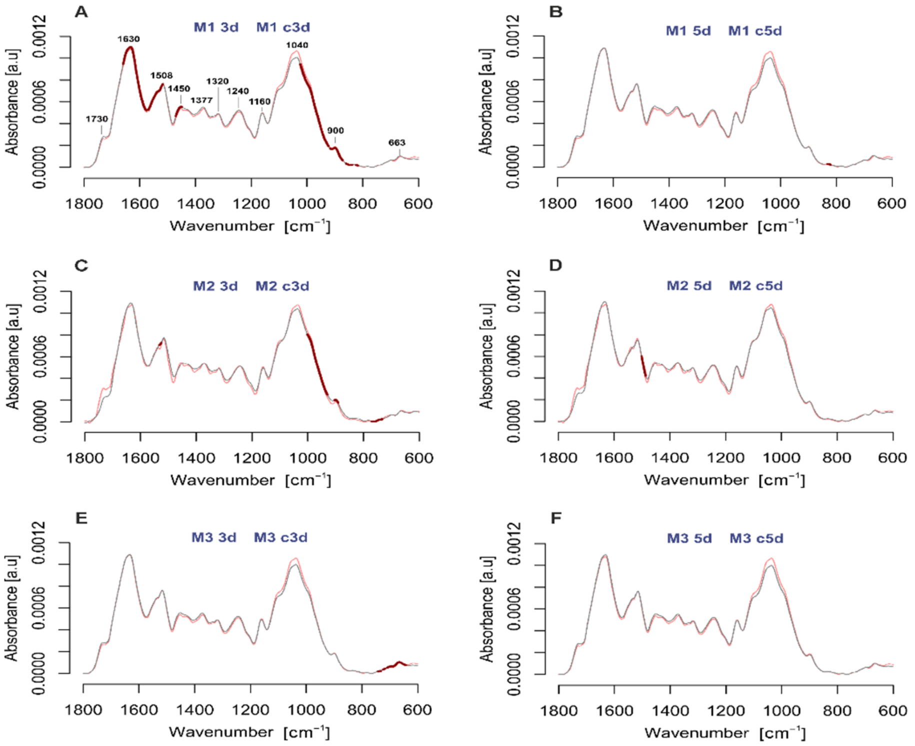

Figure S1. Mean infrared spectra collected from cell wall material for the control (grey) and chilled (red) plants of three *Miscanthus* genotypes. Comparisons were made for each of *M. giganteus* "Bydgoszcz", M1 (**A**,**B**), *M. giganteus* "Radzików", M2 (**C**,**D**), *M. giganteus* "Majdan Sieniawski", M3

(**E,F**) between the control (c3d, c5d) and chilled plants (3d, 5d). Spectral regions indicating significant changes as a result of chilling are depicted by thick red lines. The significance ($p < 0.05$) of the treatment was derived from planned contrasts based on a three-way ANOVA model and was calculated for each wavenumber within 1800-600 cm^{-1} region. The "holm" correction was used for the multiplicity adjustment.
